# Numerical evaluation of laminar heat transfer enhancement in nanofluid flow in coiled square tubes

**DOI:** 10.1186/1556-276X-6-376

**Published:** 2011-05-09

**Authors:** Agus Pulung Sasmito, Jundika Candra Kurnia, Arun Sadashiv Mujumdar

**Affiliations:** 1 Department of Mechanical Engineering, National University of Singapore, 9 Engineering Drive 1, Singapore, 117576 Singapore; 2 Minerals, Metals and Materials Technology Centre, National University of Singapore, 9 Engineering Drive 1, Singapore 117576 Singapore

## Abstract

Convective heat transfer can be enhanced by changing flow geometry and/or by enhancing thermal conductivity of the fluid. This study proposes simultaneous passive heat transfer enhancement by combining the geometry effect utilizing nanofluids inflow in coils. The two nanofluid suspensions examined in this study are: water-Al_2_O_3 _and water-CuO. The flow behavior and heat transfer performance of these nanofluid suspensions in various configurations of coiled square tubes, e.g., conical spiral, in-plane spiral, and helical spiral, are investigated and compared with those for water flowing in a straight tube. Laminar flow of a Newtonian nanofluid in coils made of square cross section tubes is simulated using computational fluid dynamics (CFD)approach, where the nanofluid properties are treated as functions of particle volumetric concentration and temperature. The results indicate that addition of small amounts of nanoparticles up to 1% improves significantly the heat transfer performance; however, further addition tends to deteriorate heat transfer performance.

## Introduction

Convective heat transfer can be enhanced by active as well as passive methods. While the former usually provide better enhancement, it requires additional external forces and/or equipment which can increase the complexity, capital, and operating costs of the system. In contrast, passive heat transfer enhancement can be achieved by changing flow geometry or modifying thermo-physical properties of working fluid. Hence, it is generally a more desirable approach when compared to an active method. In our previous study [1-3] (Sasmito AP, Kurnia JC, Mujumdar AS: Numerical evaluation of transport phenomena in a T-junction micro-reactor with coils of square cross section tubes, submitted), we have shown that coiled tubes provide better heat transfer performance relative to straight tubes under certain conditions. In this study, the potential application of coiled tubes using nanofluids to improve heat transfer performance is investigated.

Coiled tubes have been known as one of the passive heat transfer enhancement techniques in heat and mass transfer applications due to the presence of secondary flows which improve heat and mass transfer rates. They have been widely used in process industries, e.g., heat exchangers and chemical reactors, due to their compact design, high heat transfer rate, and ease of manufacture. Aside from their industrial applications, studies of the transport phenomena in coiled duct have also attracted many attention from engineering researchers. The presence of secondary flows induced by coil curvature and the complex temperature profiles caused by curvature-induced torsion are among significant phenomena which can be observed in coiled tubes. Numerous experimental [4-8] and numerical [1-3,9-13] investigations on heat transfer and flow characteristics inside coiled tubes have already been reported. Furthermore, reviews on the flow and heat transfer characteristics and potential application of coiled tubes in process industries and heat transfer application can be found in [14,15].

It is well known that conventional heat transfer fluids including water, oil, and ethylene glycol mixtures have poor heat transfer rate due to their low thermal conductivity. Therefore, over the past decade, extensive research have been conducted to improve thermal conductivity of these fluids by suspending nanoparticles of diverse materials in heat transfer fluids, called nanofluids [16]. Modern technology provides opportunities to process and produce particles below 50 nm. It is also expected that nanofluids should provide not only higher heat transfer rate, but also good stability of the suspension by eliminating possible agglomeration and sedimentation to permit long-term application [17]. To date, several experimental (see for example [18-23]) and numerical (see for example [24-28]) investigations to characterize heat transfer performance of nanofluids have been already reported. Choi et al. [18] showed that addition of small amounts of less than 1% nanoparticles can double the thermal conductivity of working fluids. Vajjha et al. [24] showed that heat transfer rate increases up to 94% by adding 10% Al_2_O_3 _nanofluid and increase up to around 89% by adding 6% CuO nanofluid. In addition, the comprehensive reference on nanofluids can be found in the book of Das et al. [29], while several reviews of nanofluids are available in the literature [30-42].

It has been shown that coiled tubes geometry and nanofluids can passively enhanced heat transfer performance. Now, to maximize the advantages of the heat transfer enhancement, we propose to combine both techniques simultaneously; i.e., employing the combination of coiled tubes filled with nanofluids. Therefore, the aim of the study presented here is threefold: (i) to investigate the heat transfer performance of various configurations of coils of square tubes, e.g., conical spiral, in-plane spiral, and helical spiral, relative to the straight pipe; (ii) to evaluate simultaneous passive heat transfer enhancement-channel geometry and fluid thermo-physical properties-in coiled tubes filled with nanofluids; (iii) to study the heat performance of two different nanofluids, water-Al_2_O_3 _and water-CuO, in coiled tubes at various nanoparticle concentrations. The most significant aspect of this study is to determine the potential advantages and limitations of heat transfer enhancement of coiled of square tubes filled with nanofluids and provide design guidelines for their applications through mathematical modeling.

The layout of the article is as follows. First, the mathematical model is introduced; it comprises conservation equations for mass, momentum, and energy. The nanofluid thermo-physical properties are treated as functions of particle volumetric concentration and temperature. The mathematical model is then solved numerically utilizing finite-volume-based CFD software Fluent 6.3.26, the User-Defined Function written in C language is used extensively to capture the nanofluid properties. The model is further validated against experimental data by Anoop et al. [19] in terms of heat transfer performance for both base-fluid and nanofluid. Fluid flow and heat transfer performance of various coiled tube designs filled with nanofluids is evaluated in terms of a figure of Merit Defined later. Parametric studies for particle concentration and nanofluid type are then carried out. Finally, conclusions are drawn and possible extensions of the study are highlighted.

## Mathematical model

**Figure 1:**
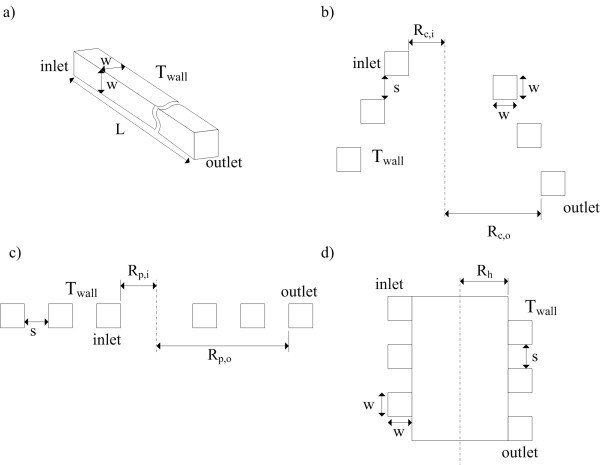
**Schematic representation of (a) straight tube, (b) conical spiral tube, (c) in-plane spiral tube, and (d) helical spiral tube**.

The physical model (see Figure 1) comprises four tube designs, e.g., straight pipe, conical spiral, in-plane spiral, and helical spiral, filled with two different nanofluids (water-Al_2_O_3 _and water-CuO). We assume that the low particle volumetric concentration of nanoparticles (less than 3%) in the base-fluid makes it behave like a single-phase fluid and there is no agglomeration or sedimentation which occurs inside the tubes. A constant wall temperature is prescribed along all sides of the channel wall; the nanofluid is assumed incompressible and Newtonian. Furthermore, to ensure fidelity of the comparison of heat transfer performance for each tube design, the total length of each tube design is kept constant. Since this study relates only to laminar flow, a precise numerical solution is adequate to simulate reality very closely.

## Governing equations

In the tube, fluid flow and convective heat transfer are taken into consideration. The con-servation equations of mass, momentum, and energy are given by [24](1)(2)(3)

In the above equations, *ρ*_nf _is the nanofluid fluid density, **u **is the fluid velocity, *p *is the pressure, *μ*_nf _is the dynamic viscosity of the nanofluid, *c*_p,nf _is the specific heat of the nanofluid and *k*_nf _is thermal conductivity of the nanofluid.

## Constitutive relations

### Thermo-physical properties of nanofluids

The thermo-physical properties of nanofluid are functions of particle volumetric concentration and temperature. The nanofluid density is given by [24,29]

where *ρ*_np _and *ρ*_w _is the nanoparticle density and water density, respectively, while *ϕ *is the particle volumetric concentration. The nanofluid viscosity is estimated by [24](5)

where  and  are constants (summarized in Table 1), and *μ*_w _is the viscosity of base-fluid.

**Table 1:** Base case and operating parameters

Parameter	Value	Unit
*c*_p,np,Al2O3_	765	J · kg^-1 ^· K
*c*_p,np, CuO_	540	J · kg^-1 ^· K
*d*_np, Al2O3_	59 × 10^-9^	m
*d*_np, CuO_	29 × 10^-9^	m
*k*_np, Al2O3_	36	W · m^-1 ^· K^-1^
*k*_np, CuO_	18	W · m^-1 ^· K^-1^
	5 × 10^4^	‾
*κ*	1.381 × 10^-23^	J · K^-1^
*ρ*_np, Al2O3_	3600	kg · m^-3^
*ρ*_np, CuO_	6510	kg · m^-3^
	9 × 10^-3^	kg · s^-1^
*p*_out_	101325	Pa
*T*_0_	298.15	K
*T*_in_	298.15	K
*T*_wall_	323.15	K
	2.8217 × 10^-2^	‾
	3.917 × 10^-3^	‾
	-3.0669 × 10^-2^	‾
	-3.91123 × 10^-3^	‾
(Al_2_O_3_)	0.9830	‾
(Al_2_O_3_)	12.959	‾
(CuO)	0.9197	‾
(CuO)	22.8539	‾
*β*_1 _(Al_2_O_3_)	8.4407	‾
*β*_2 _(Al_2_O_3_)	-1.07304	‾
*β*_1 _(CuO)	9.881	‾
*Β*_2 _(CuO)	-0.9446	‾

The specific heat of nanofluid is assumed to be a weighted average of the base-fluid and the nanoparticles, e.g.,(6)

where *c*_p,np _and *c*_p,w _are the specific heats of nanoparticle and water, respectively. In this model, the thermal conductivity considers a combination of the static part of Maxwell's theory and the dynamic part taking the contribution of the Brownian motion of nanoparticles, defined as [24](7)

where *d*_np _is the nanoparticle diameter,  is the Brownian motion constant, *k*_np _and *k*_w _are thermal conductivity of nanoparticle and water, respectively. Here, the effect of temperature and particle volumetric concentration is taken into account in the Brownian motion from empirical data given by [24](8)(9)

where *β*_1_, *β*_2_, , ,  and , are constants (see Table 1).

### Thermo-physical properties of base-fluids

The base-fluid considered in this article is water. Thermo-physical properties of water were obtained as polynomial functions of temperature [43]; the water density is defined by(10)

while the water viscosity is given by(11)

and the thermal conductivity of water is calculated from(12)

The specific heat of water is considered constant at(13)

Properties of nanoparticles are given in Table 1.

### Heat transfer performance

The heat transfer performance of the cooling channel is discussed in terms of the figure of merit, FoM, which is defined as(14)

where *W*_pump _is the pumping power required to drive the fluid flow through the channel. It is given by(15)

Here, *η*_pump _is the pump efficiency (assumed to be 70%), *W *is the total heat transfer rate, and Δ*p *is the pressure drop in the cooling channel. The total heat transfer rate is given as(16)

where  is the mass flow rate and *T*_m,in _and *T*_m,out _are mixed mean temperature at the inlet and outlet, respectively. The mixed mean temperatures is calculated as(17)

where *A*_c _is the cross section area of the channel and *V *is the mean velocity given by(18)

### Boundary conditions

The boundary conditions for the flow inside the channel are prescribed as follows

• *Inlet *At the inlet, we prescribe inlet mass flow rate and inlet temperature.(19)

• *Outlet *At the outlet, we specify the pressure and streamwise gradient of the temperature is set to zero; the outlet velocity is not known a priori but needs to be iterated from the neighboring computational cells.(20)

• *Walls *At walls, we set no slip condition for velocities and constant wall temperature.(21)

In this article, a constant mass flow rate at a Reynolds number (*Re *= *ρUD*_h_/*μ*) of approximately 1000 is prescribed at the inlet for comparison purposes.

## Numerics

**Figure 2:**
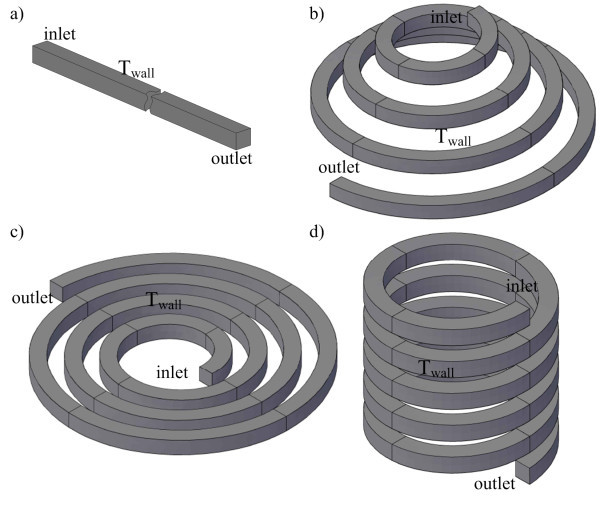
**Computational domain for (a) straight tube, (b) conical spiral tube, (c) in-plane spiral tube, and (d) helical spiral tube**.

The computational domains (see Figure 2) were created in AutoCAD 2010; the commercial pre-processor software GAMBIT 2.3.16 was used for meshing, labeling boundary conditions and determines the computational domain. Three different meshes, 1 × 10^5^, 2 × 10^5^, and 4 ×10^5^, were tested and compared in terms of the local pressure, velocities, and temperature to ensure a mesh independent solution. It is found that mesh number of around 2 × 10^5 ^gives about 1% deviation compared to mesh size of 4 × 10^5^; whereas the results from mesh number of 1 × 10^5 ^deviate by up to 8% compared to those from the finest one. Therefore, a mesh of around 2 × 10^5 ^(20 × 20 × 500) elements was considered sufficient for the numerical investigation purposes; a fine structured mesh near the wall to resolve the boundary layer and an increasingly coarser mesh in the middle of the channel to reduce the computational cost.

Equations 1-3 together with appropriate boundary conditions and constitutive relations comprising of five dependent variables, *u*, *v*, *w*, *p*, and *T*, were solved using the finite volume solver Fluent 6.3.26. User-Defined functions (UDF) were written in C language to account for particle volumetric concentration and temperature-dependence of the thermo-physical properties of the nanofluids.

The equations were solved with the well-known Semi-Implicit Pressure-Linked Equation (SIMPLE) algorithm, first-order upwind discretization and Algebraic Multi-grid (AMG) method. As an indication of the computational cost, it is noted that on average, around 200-500 iterations and 500 MB of Random Access Memory (RAM) are needed for convergence criteria for all relative residuals of 10^-6^, this takes 5-30 min on a workstation with a quad-core processor (1.83 GHz) and 8 GB of RAM.

## Results and discussion

The numerical simulations were carried out for four different tube geometries, four different nanofluid concentrations, and two different nanofluid suspensions. The base-case conditions together with the physical parameters are listed in Table 1, while the geometric details can be found in Table 2.

**Table 2:** Geometric parameters

Parameter	Value	Unit
*w*	1.00 × 10^-2^	m
*s*	1.00 × 10^-2^	m
*R*_pi_	2.00 × 10^-2^	m
*R*_po_	9.00 × 10^-2^	m
*R*_ci_	2.00 × 10^-2^	m
*R*_co_	9.00 × 10^-2^	m
*R*_h_	4.00 × 10^-2^	m
*L*	1.20	m

### Validation

When developing and implementing mathematical model to predict the behavior of nanofluid heat transfer, one needs to pay special attention to validation of the model due to inherent complexity of coupled physical phenomena and interaction between base-fluid and nanoparticle. In this study, we aim to validate our model with an experimental nanofluid heat transfer by Anoop et al. [19], which has error of approximately 4%. The heat transfer performance of nanofluid flows in circular tube with diameter 4.75 × 10^-3 ^m and length of 1.2 m is approximated with 2D axisymmetric model, see Anoop et al. [19] for details of the experimental setup.

**Figure 3:**
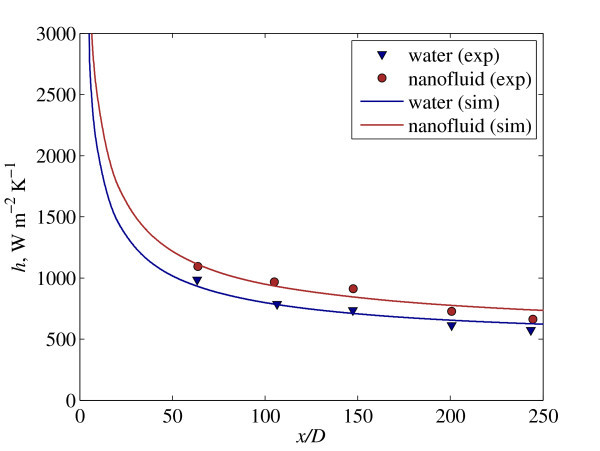
**Comparison of heat transfer coefficient between simulation (lines) and experimental data **[19]** (symbols) for water and nanofluid**.

The validation is initiated with heat transfer performance of water flowing at a constant Reynolds approximately 1580; after which, the heat transfer performance of 4 wt% of water-Al_2_O_3 _nanofluid with nanoparticle size 45 nm flows at Reynolds approximately 1588 is compared, as depicted in Figure 3. It is found that the model predictions agree well with the heat transfer performance from experimental counterpart for both water and nanofluid. This implies that the model correctly accounts for the fundamental physics associated with nanofluid heat transfer.

### Effect of geometry

#### Base-fluid

**Figure 4:**
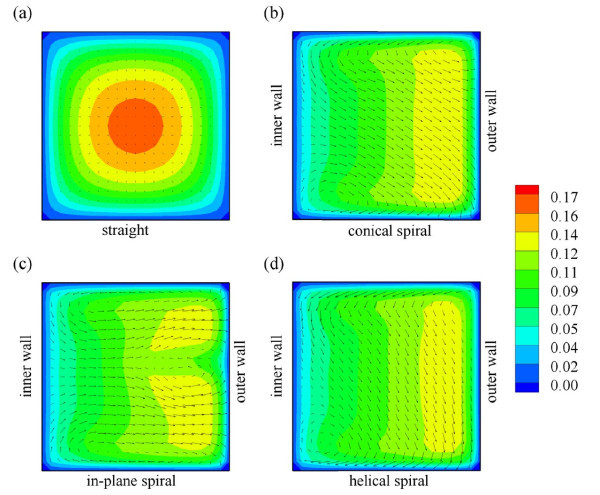
**Velocity profiles of water flow in (a) straight duct; (b) conical spiral duct; (c) in-plane spiral duct; and (d) helical spiral duct at *L *= 50 cm**.

One of the key factors that determine the heat transfer performance is the cross-sectional tube geometry. This study examines four different square cross section tubes geometries: straight, conical spiral, in-plane spiral, and helical spiral with water as the base working fluid. Since the convective heat transfer inside the tube is directly linked to flow behavior, it is of interest to investigate the flow patterns inside the tubes. In our previous studies [1-3], albeit using air as working fluid, showed that the presence of centrifugal force due to curvature leads to significant radial pressure gradients in flow core region. In the proximity the inner and outer walls of the coils, however, the axial velocity and the centrifugal force will approach zero. Hence, to balance the momentum transport, secondary flow should develop along the outer wall. This is indeed the case, as can be seen in Figure 4, where the secondary flow with higher velocities is generated in the outer wall region of coiled tubes (see Figure 4b,c,d). However, this is not the case for the straight tube (Figure 4a) as a fully developed flow exists inside the tube. It is noted that at this particular Reynolds number (approximately 1000), the secondary flows appear as one-pair for conical spiral and helical spiral tubes; whereas in the in-plane spiral tube, the secondary flows appeared as two-pairs.

**Figure 5:**
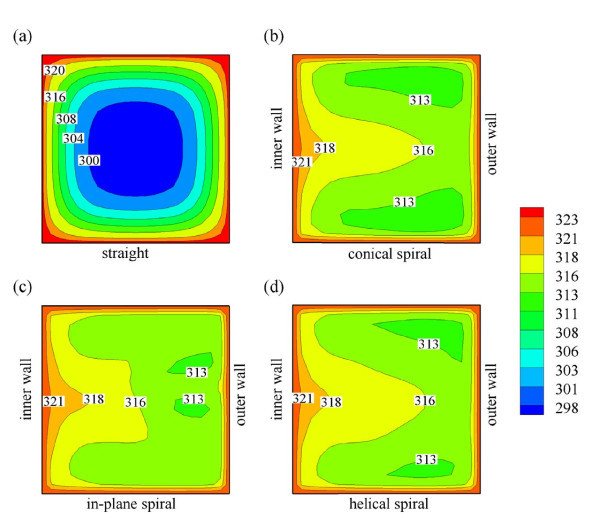
**Temperature distribution of water flow in (a) straight duct; (b) conical spiral duct; (c) in-plane spiral duct; and (d) helical spiral duct at *L *= 50 cm**.

The presence of secondary flow with high velocities is expected to have direct impact on the heat transfer rate. This can be inferred from Figure 5 which presents temperature distribution over the cross sections of various tube designs. As can be seen from Figure 5, temperatures in coiled tubes are higher than in straight tube at the same axial distance which indicates that coiled tubes have higher heat transfer rate when compared to that of the straight tube due to the presence of secondary flows. It is also worth noting that the higher intensity of secondary flow will tend to lead to higher heat transfer rate.

**Figure 6:**
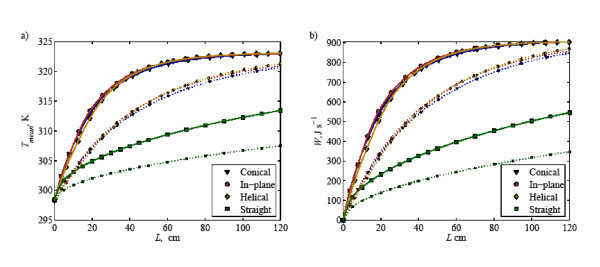
**(a) Mixed mean temperature and (b) total heat transfer at various coiled tubes along the tube length for water [⋯] and water with 1% Al_2_O_3 _[-]**.

Now looking at the mixed mean temperature and total heat transfer variation along the tube length (see dotted line in Figure 6), it is noted that coiled tubes have superior heat transfer performance when compared to that of the straight tube; the total heat transfer rate can be up to almost three times higher than that for the straight tube. In the near-inlet region, the heat transfer performance of in-plane spiral yields the best result among others, followed by conical spiral and helical spiral; whereas, in the near-outlet region, the helical coil performs the best followed by in-plane spiral and conical spiral. This indicates that, for water as working fluid, in-plane spiral is more effective to be used in short tube applications, while the helical spiral is more effective for long tube applications in terms of amount of heat transferred.

#### Nanofluids

Four square cross section tube geometries were examined for flow of nanofluid suspensions of water-Al_2_O_3 _with nanoparticle concentration of 1%. The results are depicted in Figure 6 where the mixed mean temperature and total heat transfer of base-fluid and nanofluids are shown. It is noted that adding 1% concentration of Al_2_O_3 _in water improves the heat transfer performance. The total heat transfer for straight tube increases up to 50% as compared to that for water, whereas for coiled tubes, the heat transfer improves by about 50% in the near-inlet region and then decreases toward the outlet. Furthermore, among the coiled tube geometries, in-plane spiral gives the highest heat transfer improvement, followed by helical spiral and conical spiral tubes. This implies that in-plane spiral tube may have potential application to be used along with nanofluid due to its higher heat transfer performance. Therefore, the most of the following results refer to in-plane spiral coils.

### Effect of nanoparticle concentration

**Figure 7:**
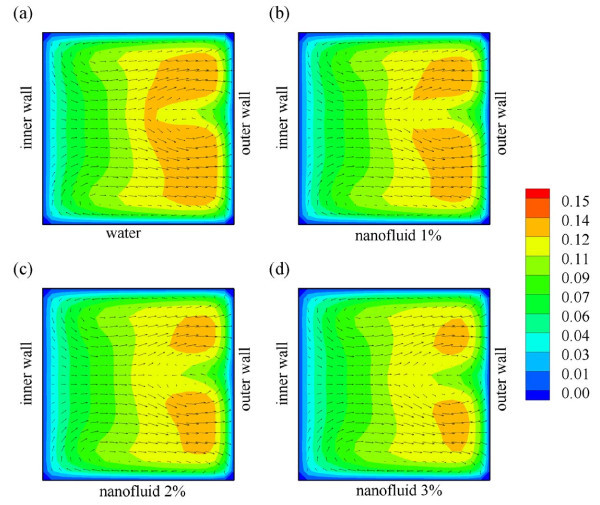
**Velocity profiles of (a) water, (b) water with 1% Al_2_O_3_, (c) water with 2% Al_2_O_3_, and (d) water with 3% Al_2_O_3 _flows inside an in-plane coiled tube at *L *= 50 cm**.

**Figure 8:**
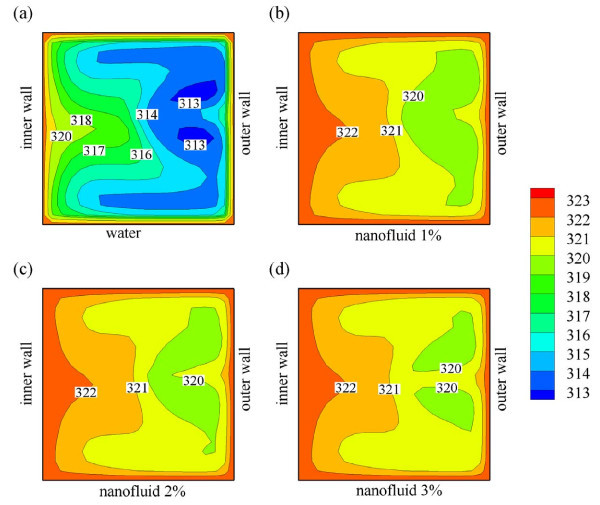
**Temperature distribution of (a) water, (b) water with 1% Al_2_O_3_, (c) water with 2% Al_2_O_3_, and (d) water with 3% Al_2_O_3 _flows inside an in-plane coiled tube at *L *= 50 cm**.

The amount of nanoparticles suspended in the base-fluid plays a significant role in deter-mining heat transfer performance. Intuitively, adding larger amount of nanoparticles in the base-fluid increases thermal conductivity of the nanofluid; however, care has to be taken as it also increases the friction factor and may reduce the stability of nanofluids due to agglomeration and sedimentation. To study the impact of these factors, we investigated four different nanoparticle concentrations: 0, 1, 2, and 3% of Al_2_O_3 _in the base-fluid (water). Figure 7 displays the velocity profiles for the in-plane spiral tube for various nanoparticle concentrations. Interestingly, the velocity profiles are not strongly affected by the additional nanoparticle suspension, especially at low concentrations. We note that at 1 and 2% of Al_2_O_3 _concentration, there is no significant difference on the secondary flow development inside the tube; whereas, at 3% Al_2_O_3 _concentration, the effect of nanofluid suspension becomes stronger: the secondary flow appears in two-pairs as compared to that in one-pair at lower nanoparticle concentrations. A plausible explanation is the fact that nanofluid suspension does not significantly change viscosity of the fluid. Conversely, this is not the case for thermal conductivity of the nanofluid, as mirrored in Figure 8, where the addition of small amount of nanoparticle (1%) drastically changes the temperature profiles inside the tube. Furthermore, the temperature profiles for higher amount of nanoparticle concentration (2 and 3%) also slightly change, but they are mainly affected by the hydrodynamics (secondary flows).

**Figure 9:**
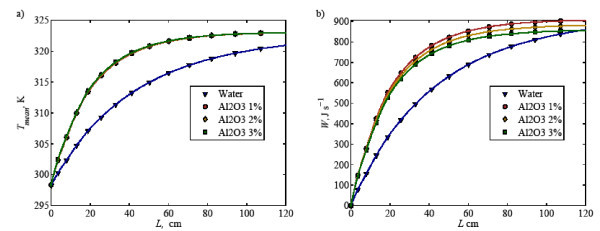
**(a) Mixed mean temperature and (b) total heat transfer at various concentrations of Al_2_O_3 _inside an in-plane coiled tube along the tube length**.

Proceeding to the local mixed mean temperature and total heat transfer along the tube, as illustrated in Figure 9, it is clearly seen that additional small amounts of nanoparticles improves the heat transfer performance significantly, especially in the near-inlet area. How-ever, increase in nanoparticle concentration leads to a reduction of total heat transfer along the tube by approximately 5%. It is noteworthy that adding large amounts of nanoparticles in the suspension is not effective in enhancing heat transfer. Moreover, low nanoparticle concentration also has advantages of better stability of the suspension as it minimizes agglomeration and sedimentation.

### Effect of nanofluid type

**Figure 10:**
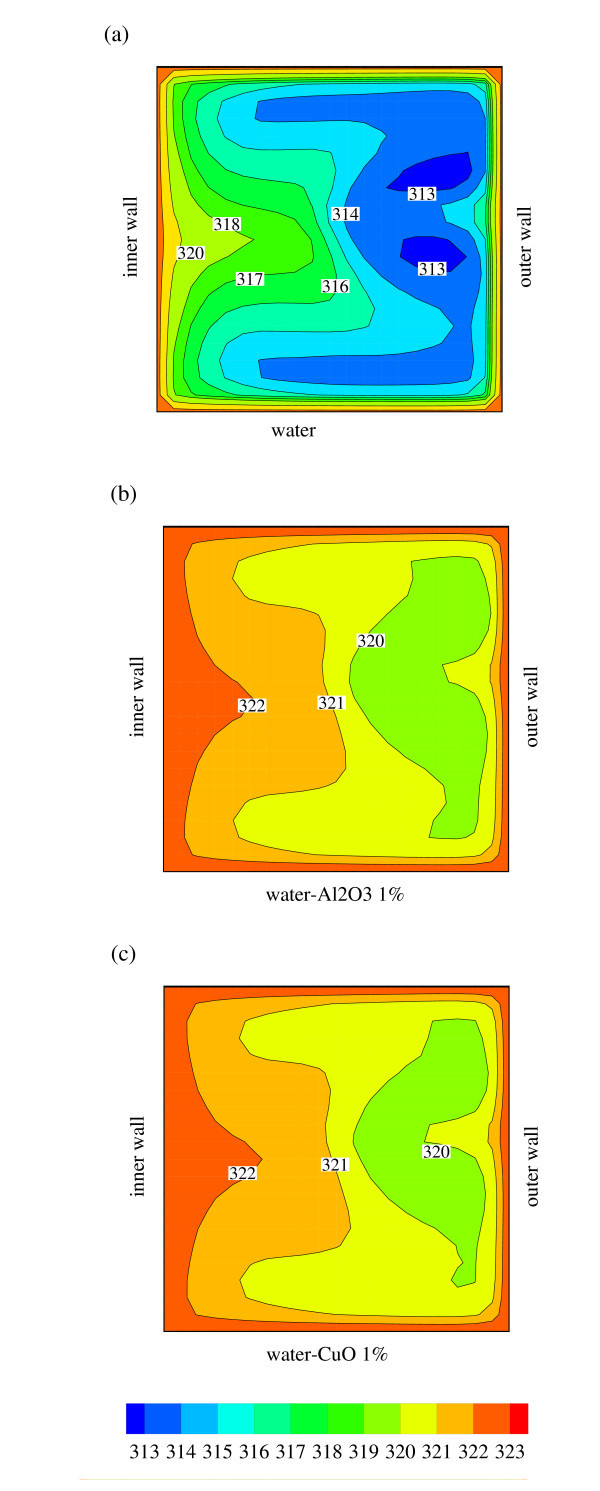
**Temperature distribution of (a) water, (b) water with 1% Al_2_O_3_, and (c) water with 1% CuO flows inside an in-plane coiled tube at *L *= 50 cm**.

So far, the simulated nanofluid type chosen was water-Al_2_O_3_; it is, therefore, of interest to see the heat transfer performance for a different nanofluid. In this study, we compare the performance of water-Al_2_O_3 _and water-CuO nanofluids. Note that other types of nanofluid suspensions can be easily simulated within the framework of this model once their properties are known. Figure 10 shows temperature profiles for an in-plane spiral tube flowing through with water (Figure 10a), 1% of Al_2_O_3 _nanofluid (Figure 10b) and 1% of CuO nanofluid (Figure 10c). We note that the temperature profiles for both nanofluids (Figure 10b,c) are much higher than that of water (Figure 10a). Closer inspection reveals that a slightly larger area of higher temperature exists for the Al_2_O_3 _suspension (Figure 10b) as compared to that for CuO suspension (Figure 10c). This is attributed to the stronger secondary flow observed in Al_2_O_3 _nanofluid when compared to that of the CuO nanofluid (not shown here due to page limitation).

**Figure 11:**
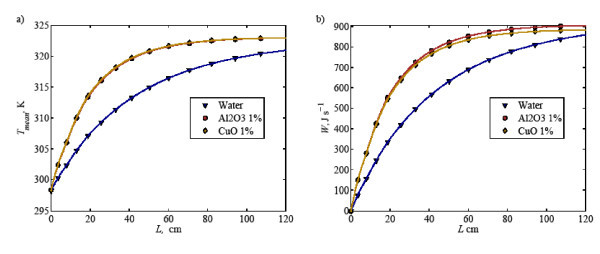
**(a) Mixed mean temperature and (b) total heat transfer of water and nanofliuds (Al_2_O_3 _and CuO) inside an in-plane coiled tube along the tube length**.

The heat transfer performance of two different nanofluid types is further evaluated in terms of the local mixed mean temperature and total heat transfer. As seen in Figure 11, the mixed mean temperature for the nanofluid is around 15% higher than that of water. There is no discernible difference between Al_2_O_3 _and CuO suspensions in terms of the mixed mean temperature. For total heat transfer, Al_2_O_3 _gives somewhat higher heat transfer (approximately 5%) when compared to the CuO nanofluid. Therefore, it can be deduced that Al_2_O_3 _nanofluid performs better heat transfer performance than that of CuO nanofluid, but not significantly. The stability and cost would decide the selection between these two nanofluids.

### Overall heat transfer performance

**Figure 12:**
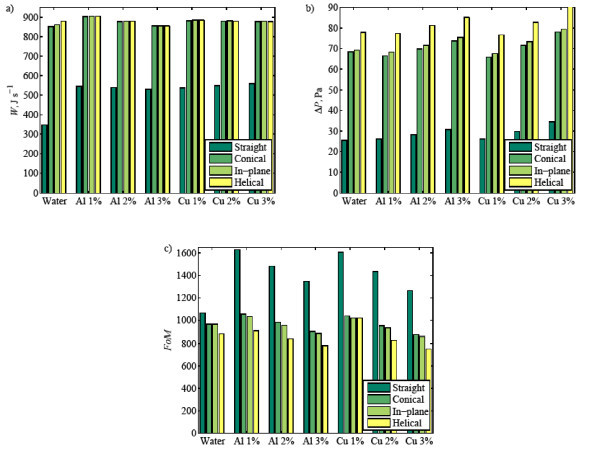
**(a) Total heat transfer, (b) pressure drop, and (c) Figure of Merit (FoM) of water and nanofluids in various tubes**.

A summary of heat transfer performance for all cases considered in this article is presented in Figure 12. Here several features are apparent; foremost among them is that the coiled tubes provide significantly higher heat transfer than that of straight tube, and addition of a small amount of nanoparticles in the base-fluid enhances heat transfer further (see Figure 12a). It is noted that the maximum heat transfer performance is achieved at 1% nanoparticle concentration, decreasing with higher amounts of nanoparticles.

Aside from higher heat transfer performance, keeping pressure drop at a minimum is of interest for reducing the operating cost and saving energy. Figure 12b shows a summary of the pressure drop required for all cases studied. Note that the mass flow rate is kept constant in all cases; hence, it can be used directly to represent the pumping power required. The straight channel requires the lowest pressure drop among all cases; whereas the coiled tube designs require more than double the pressure drop of the straight channel. Among the coiled tubes, helical spiral tube needs the highest pressure drop, followed by in-plane spiral and conical spiral tubes. An interesting phenomenon is observed at a nanofluid concentration of 1% when the pressure drop for coiled tubes is slightly lower than that for water. This is due to the fact that at low particle concentrations, the particle volumetric concentration affects the nanofluid viscosity negligibly while the effect of temperature increases in the nanofluid thermo-physical properties.

With respect to the heat transfer performance and pressure drop required in the system, the "Figure of Merit" concept is introduced as a measure of the heat transferred per unit pumping power (see Equation 14 for details). Figure 12c presents the computed figures of merit for various tube geometries, nanofluid concentrations and nanofluid types. It is found that apart from the higher heat transfer rate, the coiled tubes have lower figures of merit than those of the straight tube. This can be explained by the higher pressure drops required in the coiled systems (see Figure 12b). Among all coiled tubes tested, the conical spiral tube gives the highest figure of merit, followed by in-plane spiral and conical spiral tubes. Furthermore, for the straight tube, the addition of nanoparticles improves the figure of merit significantly, albeit it decreases with increasing concentration. For coiled tubes filled with nanofluids, on the other hand, the improvement of figure of merit is only shown at low particle concentration of 1% and then it drops lower than that of water when more nanoparticles are added. Clearly, these results suggest that one can add nanoparticle up to 1% volumetric concentration to water to enhance heat transfer performance in coiled tubes; higher nanoparticle concentrations are not recommended.

## Concluding remarks

A computational study was conducted to investigate the laminar flow heat transfer performance of square cross section tubes, i.e., straight, conical spiral, in-plane spiral, and helical spiral, with water and two nanofluids. It is found that adding 1% nanoparticle volumetric concentration improves heat transfer performance and the figure of merit for all tubes. However, higher amounts of nanoparticles is not recommended. In-plane spiral tubes give better performance than other coiled tubes for nanofluids. Furthermore, Al_2_O_3 _nanofluid gives slightly better heat transfer performance than CuO nanofluid in coiled tubes. Future study will evaluate various modeling approaches for nanofluid heat transfer, e.g., single-phase, two-phase mixture, Euler-Euler, and Euler-Lagrange models, in coils with respect to the effect of secondary flow to the nanoparticle concentration.

## Abbreviations

AMG: algebraic multi-grid; CFD: computational fluid dynamics; RAM: random access memory; SIMPLE: semi-implicit pressure-linked equation; UDF: user-defined functions. **List of symbols**: *A*_c_: Cross section area (m^-2^); *c*_p_: Specific heat (J · kg^-1 ^· K^-1^); : Viscosity parameter; *d*_p_: Particle diameter (m); *D*_h_: Hydraulic diameter (= 4*A*_c_/*P*_c_) (m); FoM: Figure of merit; *h*: Heat transfer coefficient (W · m^-2 ^· K^-1^); *k*: Thermal conductivity (W · m^-1 ^· K^-1^); *κ: *Boltzmann constant (J · K^-1^); : Brownian motion constant; *L*: Total length channel (m); : Mass flow rate (kg · s^-1^); *p*: Pressure (Pa); *P_c_*: Cross section perimeter (m); *R*: Radius of coil (m); *Re*: Reynolds number (= *ρU D*_h_/*μ*); *s*: Spacing (m); *T*: Temperature (K); **u**, *u*, *v*, *w*, *U*: Velocity (m · s^-1^); *V*: Mean velocity (m · s^-1^); *w*: Channel width; *W: *Total heat transfer (J · s^-1^); *W*_pump_: Pumping power (W). **Greek**: *β*: Brownian motion parameter; *ρ*: Fluid density (kg · m^-3^); *ϕ*: Particle volumetric concentration (%); *η*: Efficiency (%); *μ*: Dynamic viscosity (Pa · s). **Subscripts**: c: Conical spiral; h: Helical spiral; i: Inner; in: Inlet; L: Length; mean: Mean value; norm: Normalized value; nf: Nanofluids; np: Nanoparticle; o: Outer; out: Outlet; p: In-plane spiral; pump: Pump; w: Water; wall: Wall.

## Competing interests

The authors declare that they have no competing interests.

## Authors' contributions

APS developed the mathematical model together with JCK, built computational code, carried out the numerical simulation and writing the manuscript. JCK prepared created the computational domain, conducted post-processing and participated in preparing manuscript. Both APS and JCK performed the analysis. ASM supervised the whole work and edited the manuscript. All authors read and approved the final manuscript.
